# Idiopathic Scoliosis Families Highlight Actin-Based and Microtubule-Based Cellular Projections and Extracellular Matrix in Disease Etiology

**DOI:** 10.1534/g3.118.200290

**Published:** 2018-06-21

**Authors:** Erin E. Baschal, Elizabeth A. Terhune, Cambria I. Wethey, Robin M. Baschal, Kandice D. Robinson, Melissa T. Cuevas, Shreyash Pradhan, Brittan S. Sutphin, Matthew R. G. Taylor, Katherine Gowan, Chad G. Pearson, Lee A. Niswander, Kenneth L. Jones, Nancy H. Miller

**Affiliations:** * Department of Orthopedics, University of Colorado Anschutz Medical Campus, Aurora, CO; † Musculoskeletal Research Center, Children’s Hospital Colorado, Aurora, CO; ‡ Department of Cardiology, University of Colorado Anschutz Medical Campus, Aurora, CO; § Department of Pediatrics, University of Colorado Anschutz Medical Campus, Aurora, CO; ** Department of Cell and Developmental Biology, University of Colorado Anschutz Medical Campus, Aurora, CO; †† Department of Molecular, Cellular and Developmental Biology, University of Colorado Boulder, Boulder, CO

**Keywords:** idiopathic scoliosis, exome sequencing, actin cytoskeleton, microtubules, cilia, extracellular matrix

## Abstract

Idiopathic scoliosis (IS) is a structural lateral spinal curvature of ≥10° that affects up to 3% of otherwise healthy children and can lead to life-long problems in severe cases. It is well-established that IS is a genetic disorder. Previous studies have identified genes that may contribute to the IS phenotype, but the overall genetic etiology of IS is not well understood. We used exome sequencing to study five multigenerational families with IS. Bioinformatic analyses identified unique and low frequency variants (minor allele frequency ≤5%) that were present in all sequenced members of the family. Across the five families, we identified a total of 270 variants with predicted functional consequences in 246 genes, and found that eight genes were shared by two families. We performed GO term enrichment analyses, with the hypothesis that certain functional annotations or pathways would be enriched in the 246 genes identified in our IS families. Using three complementary programs to complete these analyses, we identified enriched categories that include stereocilia and other actin-based cellular projections, cilia and other microtubule-based cellular projections, and the extracellular matrix (ECM). Our results suggest that there are multiple paths to IS and provide a foundation for future studies of IS pathogenesis.

Idiopathic scoliosis (IS) is a common disorder of the immature skeleton that affects up to 3% of the pediatric population, with girls often more severely affected than boys (Asher and Burton 2006). IS is defined clinically as a structural lateral spinal curvature of ≥10° with a rotatory component, documented by radiologic analysis and occurring in otherwise healthy children. Current therapeutic options are limited to physical therapy, bracing, and surgery. The only treatment option for severe progressive curves is an operative spinal instrumentation and fusion, a costly procedure with life-long implications. IS is known to have a genetic component, with higher concordance rates in monozygotic twins than in dizygotic twins and an increased risk for first-degree relatives compared to the general population (Cowell *et al.* 1972; Riseborough and Wynne-Davies 1973; Bonaiti *et al.* 1976; Czeizel *et al.* 1978; Kesling and Reinker 1997; Inoue *et al.* 1998; Aksenovich *et al.* 1999; Andersen *et al.* 2007; Ward *et al.* 2010; Tang *et al.* 2012).

Genetic studies for IS have been conducted for more than 30 years. Genome-wide linkage studies have resulted in the identification of large chromosomal regions rather than specific genes (Salehi *et al.* 2002; Chan *et al.* 2002; Justice *et al.* 2003; Miller *et al.* 2005; Gao *et al.* 2007; Ocaka *et al.* 2008; Gurnett *et al.* 2009; Raggio *et al.* 2009; Edery *et al.* 2011). Several genome-wide association studies (GWAS) in different ethnic populations have identified potential genes and regions of interest (Sharma *et al.* 2011; Takahashi *et al.* 2011; Kou *et al.* 2013; Miyake *et al.* 2013; Ogura *et al.* 2015; Zhu *et al.* 2015; Sharma *et al.* 2015; Chettier *et al.* 2015; Zhu *et al.* 2017; Ogura *et al.* 2017). Both the *LBX1* (Takahashi *et al.* 2011; Fan *et al.* 2012; Jiang *et al.* 2013; Gao *et al.* 2013; Londono *et al.* 2014; Chettier *et al.* 2015; Grauers *et al.* 2015; Zhu *et al.* 2015; Guo *et al.* 2016; Cao *et al.* 2016; Liu *et al.* 2017; Nada *et al.* 2018) and *GPR126* (Kou *et al.* 2013; Xu *et al.* 2015; Karner *et al.* 2015; Qin *et al.* 2017) genes have been associated with IS in multiple studies. LBX1 is a transcription factor and core regulator of myoblast development (Masselink *et al.* 2017), while GPR126 functions within Schwann cells to control differentiation and myelination (Mogha *et al.* 2013). Recently, exome sequencing has identified genes that may contribute to the IS phenotype, including genes that encode components of the extracellular matrix (ECM), cytoskeleton, cilia, and centrosomes (Buchan *et al.* 2014a; Baschal *et al.* 2014; Patten *et al.* 2015; Li *et al.* 2016; Oliazadeh *et al.* 2017; Einarsdottir *et al.* 2017; Haller *et al.* 2016). Overall, these genetic findings lead to proposed mechanisms of IS pathogenesis stemming from alterations in the ECM, cilia, muscle, and axon myelination. Even with these findings, the biological basis of IS is not well understood and the genetic contributions are still poorly defined.

With the discovery of multiple genes that may contribute to IS etiology, it has become apparent that IS is likely a polygenic disease (Kruse *et al.* 2012; Haller *et al.* 2016). In this study, we completed exome sequencing in five multigenerational families to identify variants present in all affected family members, and then used Gene Ontology (GO) term enrichment analyses of these variant lists to identify functional categories that we predict are important for IS etiology.

## Materials and Methods

### Subjects

Individuals were enrolled as described previously (Baschal *et al.* 2014). A diagnosis of IS required a standing anteroposterior spinal radiograph showing ≥10° curvature by the Cobb method with pedicle rotation, and no congenital deformity or other co-existing genetic disorder (Shands and Eisberg 1955; Kane 1977; Armstrong *et al.* 1982). If two or more individuals in a family were affected with IS using these criteria, those individuals were classified as having familial IS.

Written informed consent was obtained from study subjects who were enrolled in accordance with protocols approved by the Johns Hopkins School of Medicine Institutional Review Board and the University of Colorado Anschutz Medical Campus Institutional Review Board (Colorado Multiple Institutional Review Board, Study #06-1161 and 07-0417). All procedures involving human participants were performed in accordance with the ethical standards of these institutional review boards, the 1964 Declaration of Helsinki and its later amendments, or comparable ethical standards.

We collected blood samples from all participants and extracted genomic DNA using the QIAGEN Gentra Puregene Blood Kit or standard phenol-chloroform purification protocols (Sambrook *et al.* 1989; Moore and Dowhan 2002). In the event a blood sample was not available, a saliva sample was collected using the Oragene OGR-250 kit and extracted according to the manufacturer’s protocol.

Five multigenerational IS families with European ancestry were selected for exome sequencing. These families were selected based on the number of affected individuals in a family, scoliosis curve severity, and the degree of relationship to the proband. Where possible, distantly related individuals in each family were selected for exome sequencing.

Exome sequencing was completed for three to four individuals from each family, for a total of 16 sequenced individuals from these five families. Clinical details and family relationships are presented in Table 1, and pedigrees are provided in File S1. In some cases, pedigrees were simplified to protect the identity of the study participants. Individuals selected for exome sequencing were affected with IS, with the exception of one individual from Family B. In this family, we sequenced one unaffected individual (II-2) who is expected to carry any variants that contributed to the IS phenotype within the family. In addition to her grandchildren who were sequenced in the study (IV-3 and IV-5), her father (I-1), brother (II-5), and two nieces (III-6 and III-7) are affected with IS, but DNA was not available for these individuals.

**Table 1:** Clinical information for subjects who underwent exome sequencing. Five families underwent exome sequencing, with three to four individuals sequenced in each family. Gender, curve measurement (Cobb angle), relationship to proband, degree relationship to proband, and sequencing coverage across the exome are presented for each individual. Double curves are represented by a slash. Pedigrees are provided in Supplemental File 1

Family	Individual	Gender	Curve (Degrees)	Relationship to Proband	Degree Relationship to Proband	Coverage
A	III-4	F	52/81	proband	proband	91X
A	III-5	F	76/65	sister	1^st^ degree	51X
A	III-1	M	25	half cousin (paternal)	4^th^ degree	51X
A	II-4	F	unknown	aunt (paternal)	2^nd^ degree	68X
B	IV-3	F	34/28	proband	proband	36X
B	IV-5	M	56	cousin (maternal)	3^rd^ degree	77X
B	II-2	F	0	grandmother (maternal)	2^nd^ degree	63X
C	IV-1	F	36/20	proband	proband	49X
C	III-6	F	45	first cousin once removed (maternal)	4^th^ degree	52X
C	IV-2	M	35/70	second cousin (maternal)	5^th^ degree	38X
D	III-1	F	32/47	proband	proband	52X
D	III-5	F	78/42	cousin (paternal)	3^rd^ degree	44X
D	II-5	F	12/8	aunt (paternal)	2^nd^ degree	49X
E	IV-1	F	50/60	proband	proband	55X
E	III-3	F	50	aunt (paternal)	2^nd^ degree	40X
E	II-3	F	22/35	great aunt (paternal)	3^rd^ degree	38X

### Exome Sequencing

Exome capture was completed using 1 µg of genomic DNA from 16 individuals across five families using the Illumina TruSeq Exome kit. Samples were sequenced with a 2 × 100 bp run on the Illumina HiSeq 2000 at the University of Colorado Denver Genomics and Microarray Core Facility with three samples multiplexed per lane.

### Bioinformatic Filtering

The reads were aligned to the human genome assembly GRCh38 using GSNAP (Genomic Short-read Nucleotide Alignment Program, version 2014-12-17) (Wu and Nacu 2010) and variants were identified by FreeBayes (v1.0.1-2-g0cb2697) (Garrison and Marth 2012). The candidates were filtered by SnpEff (version 4.1g) (Cingolani *et al.* 2012) and custom scripts to retain only non-synonymous SNPs, coding indels, and variants affecting splice sites. These were also stripped of known artifacts and variants whose frequency was greater than 5% in the ExAC database (r0.3) (Lek *et al.* 2016). If the variant was annotated in the dbNSFP database (version 3.0) (Liu *et al.* 2011; Liu *et al.* 2016), it was retained only if at least one of the prediction algorithms (SIFT, Polyphen2, LRT, MutationTaster) scored it as “damaging,” signifying that the resulting change to the protein had a predicted functional consequence.

Within each family, variants were required to be shared by all individuals that underwent exome sequencing. If any individuals in the family were missing data at the position of interest, the variant was retained in the list as long as the alleles were shared by the remaining family members. This filter eliminated variants that did not segregate with the IS phenotype in each family, which reduced the number of false positive variants in our results.

Gene values for pLI and pRec were obtained from the Functional Gene Constraint download from ExAC (fordist_cleaned_exac_r03_march16_z_pli_rec_null_data.txt, modified 2016-01-13, 5:00:00 pm) (Samocha *et al.* 2014; Lek *et al.* 2016).

### Functional Category Annotations

Functional annotations for the GO terms “actin cytoskeleton” and “microtubule cytoskeleton” listed in File S2 were obtained from DAVID on 2018-05-21. Annotations for “extracellular matrix” were obtained from the Core Matrisome list (Naba *et al.* 2012).

Cilia genes were annotated using a pipeline that initially focused on two cilia gene databases, SYSCILIA Gold Standard (http://syscilia.org [van Dam *et al.* 2013]) and the Centrosome and Cilium Database (CCDB [Gupta *et al.* 2015]). We also included annotations from two cilia-related Cellular Component GO terms (“cilium” and “microtubule cytoskeleton”). All potential cilia genes were manually investigated through searches in UniProt (The UniProt Consortium 2017) and PubMed (“cilia” + [gene name]). Genes were classified as encoding cilia proteins (“yes”) if the gene was listed in at least one of the cilia gene databases, or had published evidence localizing the protein product to the cilium. Genes were classified as potential cilia genes (“potential”) if published evidence suggested the protein interacted with the cilium, but was not confirmed to be related to cilia function. Genes were not classified as cilia genes if no published evidence suggested ciliary involvement, even if they were associated with the GO term “cilium”. Final determinations were made through review of the identified articles. Genes annotated as “stereocilia” were identified from the “stereocilium” GO term and also through review of articles identified in the literature.

### GO Term Enrichment Analyses

#### PANTHER:

Gene Ontology (GO) term analysis was completed using the PANTHER website (Mi and Thomas 2009; Mi *et al.* 2013; Mi *et al.* 2017). A combined variant list was generated that contained all of the variants that were identified in any of our five families (variants were filtered as described above). The corresponding gene names were used as the input for PANTHER (270 variants in 246 genes), which resulted in 251 PANTHER IDs. Statistical overrepresentation tests were performed on 2017-11-28 using the PANTHER website, with PANTHER overrepresentation test release 20170413 and Gene Ontology database release 2017-10-23. These tests used a Bonferroni multiple testing correction for the Cellular Component GO term class. The p-values were also obtained without Bonferroni correction and those results are indicated in the text and tables where appropriate.

#### DAVID:

DAVID 6.8 was used to identify the significant GO terms and clusters in our dataset (Huang da *et al.* 2009b, 2009a). The same input gene list was used for DAVID as for PANTHER (246 genes), which resulted in 242 DAVID IDs. Functional annotation clustering was used on our dataset with custom settings and the GOTERM_CC_All annotation category. DAVID clustering is based on the genes in the dataset of interest that are shared among the GO terms. The clustering stringency settings were relaxed from default values for this analysis (both Initial Group Membership and Final Group Membership were set to 2). Additionally, the EASE threshold was set to 0.1, to match the default threshold used by DAVID for single GO term chart analyses. The EASE score can be interpreted like a p-value. Default values were used for the other settings (Similarity Term Overlap = 3, Similarity Threshold = 0.50, Multiple Linkage Threshold = 0.5). The enrichment score for the cluster is based on the EASE score for each term member (negative log scale), where higher numbers represent clusters that are more enriched.

#### BiNGO:

The Biological Networks Gene Ontology tool (BiNGO) is an open-source Java tool and Cytoscape plugin that allows for the determination of GO terms that are significantly overrepresented in a set of genes and provides a visual representation of the results (Maere *et al.* 2005; Shannon *et al.* 2003). The same input gene list was used for BiNGO as for PANTHER and DAVID (246 genes), which resulted in 196 BiNGO IDs. An overrepresentation binomial test was used, with visualization of the overrepresented categories before correction. We downloaded the ontology file go.obo from the GO website (data-version: releases/2017-11-25, CVS version 38972) (Ashburner *et al.* 2000; The Gene Ontology Consortium 2017). The BiNGO analysis used the reference set whole annotation, with the downloaded go.obo file and namespace Cellular_component. A significance level of 0.05 was used, with no multiple testing correction, and first degree relatives of significant GO terms were added to the visualization to give a clearer view of how the significant GO terms were related. A custom color gradient was used for the nodes, where blue is more highly significant (*P* = 1.0E-03) and teal is less significant (*P* = 0.05). White represents the non-significant first degree relatives. The size of the node is proportional to the number of genes in the input dataset that are annotated to that GO term. Initially, the Prefuse Force Directed Layout was used, but the terms were subsequently manually rearranged in an attempt to minimize the overlap and to create clusters or groups of related terms. GIMP v2.8 (http://gimp.org) was used to add text labels for the overall groups.

### Availability of Data Statement

The authors affirm that all data necessary for confirming the conclusions of this article are represented fully within the article and its supplementary files, including the complete lists of filtered variants for each family. Supplemental material available at Figshare: https://doi.org/10.25387/g3.6104780.

## Results

We studied five multigenerational families of European ancestry affected with familial IS (Table 1). Affected individuals were diagnosed with IS based on radiographic findings of a spinal curvature ≥10°. Exome sequencing, followed by variant detection and filtering, was completed for 16 individuals (3 to 4 individuals per family). Sequencing coverage ranged from 36 to 91X, with an average of 53X. Filtering resulted in a list of variants for each family, where each variant was an insertion/deletion, nonsense, missense, or splice-site change with a predicted functional consequence and was present at a minor allele frequency (MAF) of 5% or less in the ExAC database. We used an MAF cut-off of 5% due to the relatively high prevalence of IS in the general population (up to 3% when defining scoliosis as a Cobb angle ≥10°). In addition, to be considered for further study, we required variants to be present in all sequenced individuals in the family, as described in detail in the Methods section.

A total of 270 variants in 246 genes were identified across the five families after this filtering process, with a range of 19 to 89 variants identified in each family (File S2). Eight genes were shared by two families (*XRRA1*, *ANKRD11*, *ANKRD30B*, *DNHD1*, *ERN2*, *MYBPC3*, *NPY4R* and *TNFRSF10C*) and no genes were shared by three or more families. Even with our stringent familial analysis that required the identified variants to be present in every sequenced family member, a large number of potentially causal variants remained in each family.

To further understand and interpret these large variant lists, we completed Gene Ontology (GO) term enrichment analyses. These analyses allowed us to investigate the hypothesis that IS is caused by variants in multiple genes, and that those genes will show enrichments of certain functional annotations or pathways. We used the programs PANTHER, DAVID, and BiNGO to complete these analyses and explore this hypothesis in relation to our data.

We completed PANTHER GO term overrepresentation analysis which allowed us to compare the number of genes in our dataset annotated with each GO term to the number expected by chance. The input was our combined gene list from all five families (246 genes). The only cellular component GO term in our dataset that passed significance with a Bonferroni multiple testing correction was “stereocilium” (*P* = 0.0209 with Bonferroni correction, *P* = 1.55E-05 without Bonferroni correction, 11.70 fold enrichment, n = 6 genes), which is an actin-based cellular projection that is important for both hearing and balance.

Two major themes emerged when we examined the other enriched GO terms that were significant without Bonferroni multiple testing correction (File S3). The prominent theme from the PANTHER results are terms related to cellular projections, which represent 18 of the top 20 most significant GO terms without Bonferroni correction. The two terms in the top 20 that are not specifically related to cellular projections are “phagocytic vesicle lumen” (*P* = 6.23E-04, 55.89 fold enrichment, n = 2 genes) and “intrinsic component of plasma membrane” (*P* = 6.20E-03, 1.59 fold enrichment, n = 32 genes). Both actin-based and microtubule-based cellular projections are included in the top 20 GO terms. Actin-based cell projections in this list include two terms related to the stereocilium and two terms related to general actin-based cellular projections. The microtubule-based cellular projection terms include seven cilia-related terms, including “ciliary part” (*P* = 4.69E-03, 2.42 fold enrichment, n = 12 genes) and “axoneme part” (*P* = 8.01E-04, 9.86 fold enrichment, n = 4 genes). The term “kinocilium” (*P* = 5.35E-03, 18.63 fold enrichment, n = 2 genes) is also present in the top 20 PANTHER list. Kinocilia are a specialized type of cilia present in vestibular hair cells, and work with stereocilia to sense and respond to spatial orientation. The term “neuron projection” (*P* = 2.15E-03, 1.87 fold enrichment, n = 25 genes) is also present in the top 20 list, and includes projections that are composed of both actin and microtubules. This cell projection theme is also carried beyond the top 20 GO terms.

The second major theme from our PANTHER results (beyond the top 20 terms) is the extracellular matrix (ECM), with specific terms of “proteinaceous ECM” (*P* = 1.36E-02, 2.28 fold enrichment, n = 10 genes) and “ECM component” (*P* = 1.62E-02, 3.44 fold enrichment, n = 5 genes). Additionally, our PANTHER results include multiple terms related to collagen and collagen trimers, which are major components of the ECM. Overall, we identified themes of related GO terms from our PANTHER results, including cellular projections and ECM.

The DAVID Functional Annotation Clustering Tool is an unbiased method for clustering significantly enriched GO terms. This tool works by identifying the genes in our dataset that are shared among the GO terms. We applied this algorithm to our data, again using the Cellular Component GO terms. DAVID identified clusters in our data that loosely correlate with stereocilium/actin-based cell projections/primary cilium (Annotation Cluster 1), extracellular matrix/collagen (Cluster 2), cell projection/cilium (Cluster 3), plasma membrane components (Cluster 4), and neuron part/synapse (Cluster 5) (Table 2 and File S4).

**Table 2:** Functional annotation clusters identified by DAVID. Functional annotation clustering analysis was completed as described in the Methods. In brief, the GOTERM_CC_All annotation category was used, with an input of the 246 genes identified in our dataset. Five clusters were identified, and higher enrichment scores represent clusters that are more enriched. Detailed results, including underlying genes for each GO term, are presented in Supplemental File 4

Cluster	Enrichment Score	GO Terms
Cluster 1	2.47084939	stereocilium, stereocilium bundle, actin-based cell projection, cluster of actin-based cell projections, brush border, nonmotile primary cilium, primary cilium, microvillus
Cluster 2	0.549760389	extracellular matrix component, complex of collagen trimers, proteinaceous extracellular matrix
Cluster 3	1.470776063	cell projection, cilium, cell projection part, ciliary part
Cluster 4	1.268556732	intrinsic component of plasma membrane, integral component of plasma membrane, plasma membrane part
Cluster 5	1.058855585	synapse, neuron part

**Figure 1:**
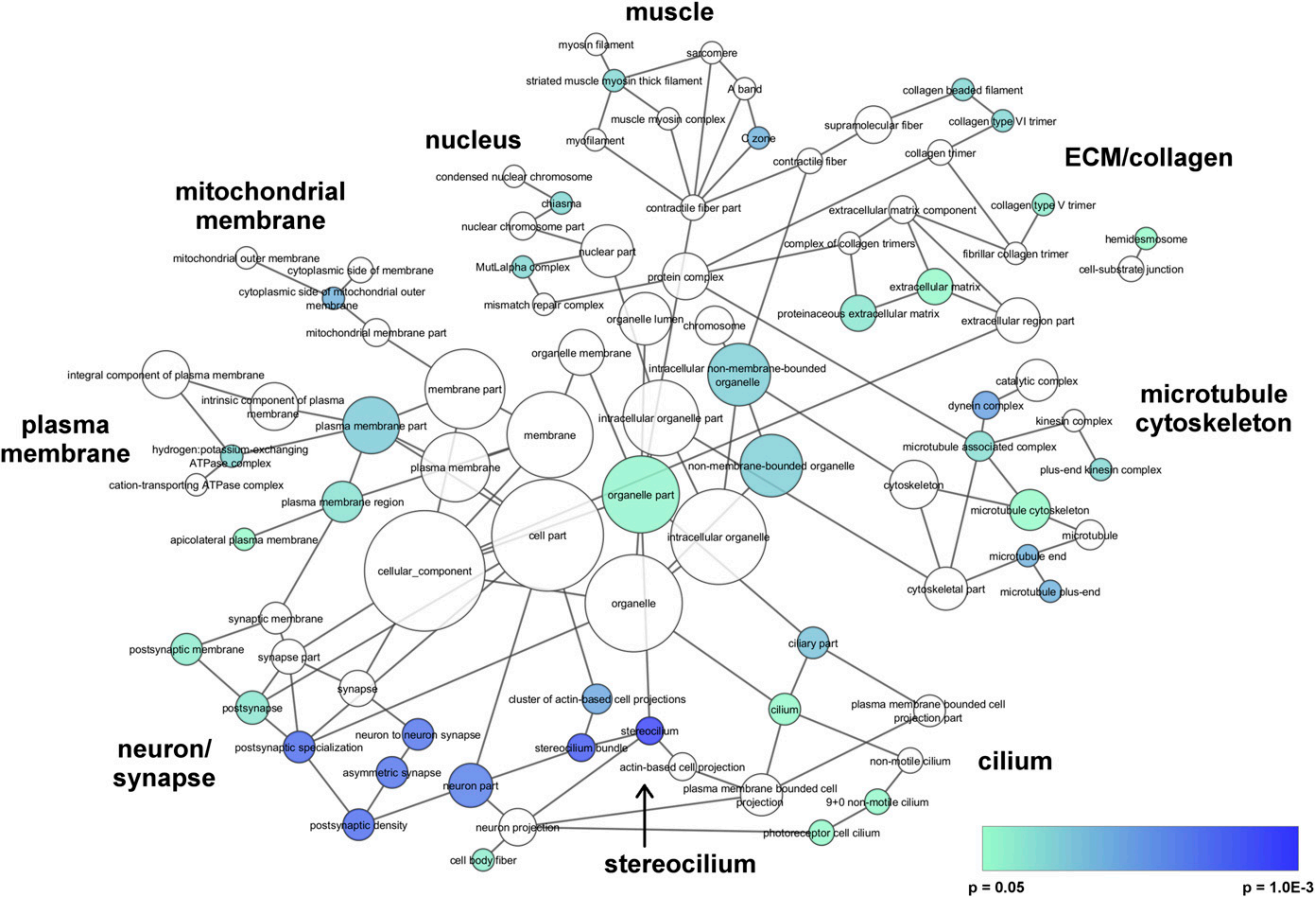
BiNGO visualization of Cellular Component GO terms. BiNGO was used to determine and visualize GO terms that are significantly overrepresented in our dataset, as described in detail in the Methods. Blue represents more significant GO terms, teal is *P* = 0.05, and white are non-significant first degree relatives. The size of the node is proportional to the number of genes in the dataset that are annotated to that GO term. The data used to generate this figure are presented in Supplemental File 6.

We used BiNGO visualization software to further investigate and understand these clusters. Overall, we found that there were nine overall “groups” of GO terms that are loosely clustered (Figure 1). The raw data are reported in File S6. We manually annotated these nine groups as GO terms related to muscle, ECM/collagen, microtubule cytoskeleton, cilium, stereocilium, neuron/synapse, plasma membrane, mitochondrial membrane, and nucleus. The genes annotated to the significant GO terms included in each of the nine overall groups are listed in Table 3. Of note, only one gene underlies the significant result in three of the nine groups (muscle, mitochondrial membrane, and nucleus). However, the significant results in groups related to our major themes (ECM/collagen, microtubule cytoskeleton, cilium, stereocilium, and neuron/synapse) are driven by at least four genes. These overall results and themes are consistent with the manual clustering we used for our PANTHER results and the algorithmic clustering used by DAVID.

**Table 3:** BiNGO GO term annotations and gene lists. BiNGO was used to determine and visualize GO terms that are significantly overrepresented in our dataset, as described in detail in the Methods and Results. Nine overall groups of clustered GO terms were manually annotated. This table lists the number of significant GO terms included in the overall group, the number of genes annotated to those significant GO terms, and the corresponding gene names. In addition, three significant GO terms were not clustered with the nine overall groups: non-membrane-bounded organelle, intracellular non-membrane-bounded organelle, and organelle part. The non-redundant list of genes annotated to those three GO terms are included, as are the genes from our five families that were not annotated by BiNGO

Overall Group	# Significant GO Terms	# Genes	Genes Annotated to the Overall Group
Muscle	2	1	*MYBPC3*
ECM/ Collagen	6	8	*COL6A3*, *SERPINA1*, *TNXB*, *FLRT2*, *DST*, *COL5A2*, *COL6A5*, *TINAG*
Microtubule Cytoskeleton	6	12	*DNAH8*, *DNAH6*, *DNHD1*, *DST*, *KIF15*, *MACF1*, *SULT1C2*, *C2CD3*, *AKAP9*, *MCM3*, *NPHP4*, *CEP290*
Cilium	4	5	*PCDH15*, *C2CD3*, *CEP290*, *DNAH8*, *NPHP4*
Sterocilium	3	4	*LOXHD1*, *PCDH15*, *USH1C*, *LRP2*
Neuron/ Synapse	7	15	*EPS8*, *CPEB1*, *DLG1*, *GRID2*, *SEMA4C*, *EPHA7*, *LRRK2*, *SEMA3A*, *CHAT*, *PCDH15*, *USH1C*, *ARID1A*, *LOXHD1*, *SACS*, *CEP290*
Plasma Membrane	4	31	*EPHB6*, *SELPLG*, *CSF3R*, *NPY4R*, *LRP2*, *AQP2*, *ATP12A*, *EPS8*, *SLC22A14*, *GNGT2*, *FLRT2*, *STAB1*, *PCDHA4*, *EPHA7*, *GRID2*, *ABCC1*, *DST*, *MGA*, *SEMA4C*, *TNFRSF10C*, *PCDHA11*, *ATRN*, *CPEB1*, *PTPRB*, *DLG1*, *GJB4*, *CCKBR*, *NEDD4*, *SCNN1A*, *SLC26A7*, *FGFR1*
Mitochondrial Membrane	1	1	*LRRK2*
Nucleus	2	1	*MLH1*
Three Significant GO Terms That Are Not in the Nine Overall Groups	3	47	*HIST1H2BJ*, *CHD9*, *NOP2*, *CHD6*, *NAT10*, *ZNF22*, *RRBP1*, *PPM1E*, *AKAP12*, *ORC4*, *MRPL3*, *CLSPN*, *LRRFIP1*, *FARP1*, *DDX11*, *KRT2*, *ELMOD3*, *DDX54*, *EBNA1BP2*, *TADA2A*, *TSR1*, *VCL*, *ECE2*, *GOLGA2*, *JPH3*, *GALNT8*, *TET1*, *CDC25A*, *ERN2*, *NDUFS8*, *MMRN1*, *MED20*, *PPIE*, *UQCRC2*, *GOLGA8A*, *POM121*, *SLC25A27*, *ATXN3*, *MAN2A2*, *UBN1*, *DHX38*, *SSR1*, *ATRIP*, *B3GAT2*, *DNAJC11*, *SON*, *QARS*
Not Annotated by BiNGO	N/A	49	*FASTKD1*, *ULK4*, *DDX60L*, *FAM216A*, *C16ORF71*, *C6ORF223*, *EVA1A*, *HENMT1*, *EPG5*, *IAH1*, *C21ORF58*, *TET2*, *RNF208*, *JOSD2*, *KIF7*, *PALD1*, *ATAD3C*, *PATL2*, *FCHSD1*, *THAP3*, *POM121L2*, *KIAA1257*, *CCDC42B*, *METTL20*, *DXO*, *NUTM2G*, *TRABD2A*, *TRUB1*, *TRIM51*, *STAMBPL1*, *DCDC2B*, *BCO1*, *C16ORF52*, *FAM83C*, *LEKR1*, *HELQ*, *FBXO39*, *TYW1B*, *DNAJC13*, *FAM111B*, *BPIFB1*, *NOL4L*, *ST5*, *KANSL3*, *R3HCC1*, *GNB3*, *CEP162*, *KBTBD8*, *CCDC173*

As cilia have been described as a potential factor in IS etiology (Grimes *et al.* 2016; Patten *et al.* 2015; Buchan *et al.* 2014b; Andersen *et al.* 2017; Einarsdottir *et al.* 2017), we further investigated the cilia connections identified in our dataset. Cilia components and functions are still under active exploration by many groups, and as a result the cilia-related GO term annotations are not always up to date. Therefore, we chose to use a different pipeline for annotating our cilia genes to generate a more inclusive list. We compared our gene list to the cilia gene databases (SYSCILIA [van Dam *et al.* 2013] and CCDB [Gupta *et al.* 2015]) and cilia-related GO terms, and also incorporated confirmatory literature searches. These annotations are included in File S2. We then used literature searches to annotate the location within the cilium for each cilia protein identified in our dataset (Table 4 and File S5). Overall, we have found that 6.3 to 8.8% of the genes identified in our five IS families encode proteins that localize to the cilium.

**Table 4:** Location of proteins in the cilium. Literature searches were used to identify the location within the cilium of the cilia-related genes/proteins identified in our study. Genes were classified as encoding cilia proteins (“yes”) if the gene was listed in at least one cilia gene database, or had published evidence localizing the protein product to the cilium (see Methods). Genes were classified as potential cilia genes (“potential”) if published evidence suggested the protein interacted with the cilium, but was not confirmed to be related to cilia function. Cilia annotations are presented for all variants in Supplemental File 2 and for cilia-localized variants in Supplemental File 5

Location	Number of Variants (“Yes”)	Number of Variants (“Potential”)
Axoneme	7	0
Basal body/centriole	2	0
Base of cilia	6	0
Other/unknown	2	7
Total	17	7

We next investigated our results at the level of individual IS families, rather than combining the results from all five families together. We compared each family’s gene list with the three functional categories identified in our analyses (stereocilia and other actin-based cellular projections, cilia and other microtubule-based projections, and the ECM, File S2). Three families had at least one gene annotated to each of these three functional categories (Families A, B, and E). Family C had genes annotated to actin cytoskeleton and microtubule cytoskeleton, but did not have an ECM gene. Family D had genes annotated to microtubule cytoskeleton and ECM, but did not have an actin cytoskeleton gene.

When these results are examined together, three themes emerge. Our IS families show enrichments of variants in genes related to stereocilia and other actin-based cellular projections, cilia and other microtubule-based projections, and the ECM. We have identified these three functional categories of genes in both the combined family dataset and in individual families with IS.

## Discussion

In this study, we report exome sequencing results for five multigenerational IS families. We identified a total of 270 variants in 246 genes across all 5 families. No genes were shared by all families, indicating that there is not a single Mendelian cause for IS within this sample group. We performed GO term enrichment analyses, with the hypothesis that certain functional annotations or pathways would be enriched in the 246 genes identified in our IS families. Using complementary programs to complete these analyses, we identified enriched categories that include stereocilia and other actin-based cellular projections, cilia and other microtubule-based cellular projections, and the extracellular matrix (ECM). These results suggest that familial IS is a polygenic disorder with multiple genes and pathways involved in the pathogenesis of the disease.

Genes related to actin-based cellular projections were the first enriched category in our dataset. These actin-based structures include stereocilia, neuronal projections, microvilli, and other actin-related genes that are not specific to cellular projections. The only GO term that passed significance with Bonferroni multiple testing correction in our data were “stereocilium” (PANTHER, *P* = 2.09E-02, n = 6 genes). Stereocilia are actin-based cellular projections that are important for both the auditory system (hearing) and the vestibular system (balance and spatial orientation). Multiple studies support an association between IS and vestibular dysfunction (Hawasli *et al.* 2015). Individuals with IS have been described to have differences in vestibular function and/or anatomy (Hawasli *et al.* 2015) and altered sensorimotor integration (Pialasse *et al.* 2015). These functional studies in individuals with IS, coupled with our genetic findings, suggest that vestibular genes may play an important role in the development of the abnormal spinal curvature that is the defining characteristic of IS.

Genes related to microtubule-based cellular projections were another enriched category in our dataset. These microtubule-based structures include cilia, neuronal projections, and other microtubule-related genes that are not specific to cellular projections. Cilia genes are enriched in our data, with significant PANTHER results for both “ciliary part” (*P* = 4.69E-03, n = 12 genes) and “axoneme part” (*P* = 8.01E-04, n = 4 genes). We found that 6.3–8.8% of the genes identified in our five IS families encode proteins that localize to the cilium, and found that all major regions of the cilium were represented (axoneme, basal body/centriole, and base of cilia). The primary cilium is a microtubule-based structure that is important for cell signaling, mechanosensation, and development, and has a significant role in the skeletal system (Anderson *et al.* 2008; Nguyen and Jacobs 2013; Temiyasathit and Jacobs 2010). Our findings are supported by several studies that suggest that ciliary dysfunction has a role in the pathogenesis of IS. Defects in motile cilia (which generate the flow of extracellular fluid) can cause a late-onset scoliosis without vertebral malformations in zebrafish (Hayes *et al.* 2014; Grimes *et al.* 2016), which was attributed to defects in cerebral spinal fluid flow (Grimes *et al.* 2016). Additional studies have also implicated basal body and cilia genes in IS etiology in humans and zebrafish (*POC5*, *kif6*, *VANGL1*, and *CELSR2*) (Patten *et al.* 2015; Buchan *et al.* 2014b; Andersen *et al.* 2017; Einarsdottir *et al.* 2017). Longer cilia were observed on osteoblasts from IS individuals when compared to controls, although the functional significance of this finding has not yet been determined (Oliazadeh *et al.* 2017). Therefore, the genetic findings from our group and others are supportive of a functional role for cilia in IS etiology.

Clinical observations also suggest a plausible role for cilia dysfunction in the pathogenesis of IS. Ciliopathies are a diverse class of human diseases that result from defects in both primary and motile cilia (Waters and Beales 2011). Importantly, ciliopathy patients, including those with Joubert, Jeune, Bardet-Biedl, Alstrӧm, and primary ciliary dyskinesia (PCD), show an increased incidence of scoliosis (Brancati *et al.* 2010; O’Brien *et al.* 2015; Ramirez *et al.* 2004; Marshall *et al.* 2011; Engesaeth *et al.* 1993; Schlösser *et al.* 2017; Knowles *et al.* 2013) Furthermore, 55 of the 303 genes from the SYSCILIA annotated list of cilia genes (van Dam *et al.* 2013) were associated with human syndromes that have clinical reports of scoliosis (Oliazadeh *et al.* 2017). This overlap suggests shared mechanisms in the etiologies of ciliopathies and IS. Additionally, cilia are crucial for both bone and cartilage development through their roles in mechanosensation, and are also responsible for directional ECM production and endochondral ossification at the growth plate (Seeger-Nukpezah and Golemis 2012). However, it is important to note that IS appears to specifically affect the spine, while most currently defined ciliopathies affect multiple tissues and organ systems. These distinct phenotypes may be explained by differences in either mutation severity or tissue-specific gene expression, which will need to be explored in functional studies.

The third functional category enriched in our data are the ECM (*P* = 1.36E-02, n = 10 genes), which is clearly evident in our BiNGO clustering (Figure 1). The ECM is a dense network of fibrous proteins that provides structural support to cells and provides tissue organization, and has been identified as important for IS etiology by multiple groups. IS patients have a significant burden of rare variants in the fibrillin genes (*FBN1* and *FBN2*) [Buchan *et al.* 2014a], which are important components of the ECM. Additionally, our group previously reported similar results for rare variants in the ECM component perlecan (*HSPG2*) [Baschal *et al.* 2014]. Finally, in a larger scale IS study, Haller *et al.* identified an increased burden of rare variants in ECM genes in IS patients, with a specific association to the musculoskeletal collagen genes. This study found that the risk of IS increases proportionally with the number of rare variants in ECM genes [Haller *et al.* 2016]. In addition, scoliosis is often a phenotypic element of diseases caused by defects in the ECM, such as Marfan syndrome and several of the Ehlers-Danlos syndromes. Therefore, our results support and expand upon the previously published literature that implicates an involvement of the ECM in IS etiology.

Actin and microtubules are both essential components of neuronal projections, and therefore neuronal projections fit into both the first and second functional categories identified from our data. The GO term “neuron projection” was significantly enriched in our dataset (*P* = 2.15E-03, n = 25 genes) and this category is evident in our BiNGO clustering (Figure 1). These genes are related to both actin-based and microtubule-based neuronal projections, encoding proteins located in the axon growth cone and dendritic spine, and also encoding proteins involved in axon guidance and neuronal growth. The axon growth cone tip and dendritic spines are actin-rich structures, while the axon growth cone central domain is a microtubule-based structure. Neuronal growth relies heavily on microtubules, and both actin and microtubules are important for the axon guidance process. Previous studies have identified neuronal genes that may be important for IS. A GWAS for IS identified significant associations near the *CHL1* and *DSCAM* genes, which both encode axon guidance proteins (Sharma *et al.* 2011). *GPR126*, replicated in multiple IS genetic studies (Kou *et al.* 2013; Xu *et al.* 2015; Karner *et al.* 2015; Qin *et al.* 2017), is important for normal myelination of axons but does not have an obvious role in the cytoskeleton (Mogha *et al.* 2013). Damaging variants in these genes may lead to mild neurological defects that give rise to IS in some patients.

IS is now believed to be a complex polygenic disease, where multiple variants, acting in a polygenic fashion, are needed to develop the phenotype. We propose two main mechanisms that could underlie the polygenic nature of IS. The first is that more than one variant in a single cellular structure or pathway could be required for IS development (*e.g.*, multiple variants in cilia genes are required in one family and multiple variants in ECM genes are required in another family). A second possibility is that a combination of variants in several cellular structures or pathways could be required for IS development (*e.g.*, at least one variant is needed in a stereocilia gene, cilia gene, and ECM gene). Our data seems support the second possibility, as three of the five families have at least one variant in each of the three enriched functional categories identified in this study (File S2). This suggests that a combination of variants in all three functional categories may be important for the development and progression of IS. This interpretation is based on only five families, and will need to be investigated further in larger familial studies. Overall, we believe that there are multiple mechanisms that can lead to IS and that our identification of variants in three enriched functional categories supports this theory.

To summarize, we identified three interconnected categories of GO terms that are enriched in our dataset of five IS families: actin-based cellular projections, microtubule-based cellular projections, and the ECM. The ECM is important for both stereocilia (actin-based cellular projections) and cilia (microtubule-based cellular projections), tying together the three functional categories identified in our dataset (Seeger-Nukpezah and Golemis 2012; Collins and Ryan 2014; Deans 2013). Previous studies support the involvement of each of these individual categories of genes and GO terms in IS etiology. Functional studies of the variants identified in these families will ultimately be needed to strengthen the link between IS and these associated genetic changes. Our data provides additional genetic support for the involvement of these functional categories in IS etiology. Once the genetic causes of IS are known, IS may be classified into multiple sub-diseases, each with a different genetic cause and different disease pathogenesis. This information will hopefully lead to further clinical insights into IS, including risk of curve progression or response to bracing, which could eventually lead to personalized disease treatments.
